# Percutaneous versus open pedicle screw instrumentation in treatment of thoracic and lumbar spine fractures: A systematic review and meta-analysis

**DOI:** 10.1097/MD.0000000000012535

**Published:** 2018-10-12

**Authors:** Feng Tian, Lai-Yong Tu, Wen-Fei Gu, En-Feng Zhang, Zhen-Bin Wang, Ge Chu, Haer Ka, Jiang Zhao

**Affiliations:** Department of Orthopedics, Traditional Chinese Medicine Hospital of Xinjiang Medical University, Urumchi, China.

**Keywords:** meta-analysis, minimally invasive surgery, open, pedicle screw fixation, percutaneous, thoracic vertebrae, thoracolumbar fractures

## Abstract

**Background:**

To assess the safety and efficacy of percutaneous short-segment pedicle instrumentation compared with conventionally open short-segment pedicle instrumentation and provide recommendations for using these procedures to treat thoracolumbar fractures.

**Methods:**

The Medline database, Cochrane database of Systematic Reviews, Cochrane Clinical Trial Register, and Embase were searched for articles published. The randomized controlled trials (RCTs) and non-RCTs that compared percutaneous short-segment pedicle instrumentation to open short-segment pedicle instrumentation and provided data on safety and clinical effects were included. Demographic characteristics, clinical outcomes, radiological outcomes, and adverse events were manually extracted from all of the selected studies. Methodological quality of included studies using Methodological Index for Non-Randomized Studies scale and Cochrane collaboration's tool for assessing the risk of bias by 2 reviewers independently.

**Results:**

Nine studies encompassing 433 patients met the inclusion criteria. Subgroup meta-analyses were performed according to the study design. The pooled results showed there were significant differences between the 2 techniques in short- and long-term visual analog scale, intraoperative blood loss, operative time, postoperative draining loss, hospital stay, and incision size, although there were no significant differences in postoperative radiological outcomes, Oswestry Disability Index, hospitalization cost, intraoperative fluoroscopy time, and adverse events.

**Conclusion:**

Percutaneous short-segment pedicle instrumentation in cases with achieve satisfactory results, could replace in many cases extensive open surgery and not increased related complications. However, further high-quality RCTs are needed to assess the long-term outcome of patients between 2 techniques.

## 1 Introduction

Fractures of the thoracic and lumbar vertebrae are quite common injuries among patients suffering from multiple traumas, and nearly one-third of patients had concomitant spinal cord injury and variable neurologic deficit.^[1,2]^ Cooper et al reported an overall incidence of 117 per 100,000 person-year who had sustained spinal injuries after high-energy accidents in young people whereas in osteoporosis was the dominant cause in elderly people.^[3]^ Those injuries are a very painful and life-affecting condition, which can impact on life quality, prolong absence from work and usually cause ongoing chronic pain. Thus, thoracolumbar fracture has already been a significant socioeconomic impact.^[4,5]^

The treatments of thoracolumbar fracture depend on the individual characteristics of the fracture that range from compression fractures and burst fractures to flexion distraction injuries with fracture dislocation, which can be managed conservatively including bed rest alone, closed reduction of fractures and functional bracing, and surgical managements involving open reduction and internal fixation of the fracture. However, some have advocated that nonoperative treatments were associated with late neurologic decline in 10% to 20% of patients and were fraught with its difficulty in moving.^[6,7]^ Posterior pedicle screw fixation is widely used in clinical practice provided 3-column fixation and biomechanically desirable as follow: enhanced rigidity and stability of the spine over conventional by maintaining anatomical alignment of the spinal column; built a multidimensional spinal fixation and more flexible system to accommodate a patient's individual anatomy; more corrected of kyphotic deformities; and in some cases, even fewer neurological risks and early painless mobilization.^[8]^ However, conventional open procedure is associated with massive blood loss, high infection rate, prolonged postoperative pain, and disability.^[6]^ In addition, this procedure has been demonstrated to cause a significant postoperative muscle atrophy and scarring which may be caused by the muscle separation lateral to the facet joints, direct trauma to the vasculature, and increased intramuscular pressure after insertion of retractors.^[9,10]^ Thus, a new perspective in the treatment of thoracolumbar fractures was offered with the development of percutaneous posterior pedicle screws procedure, which performed by sparing the paravertebral musculature and avoiding a damage to the zygapophysial joint, can also reduce bleeding, postoperative pain, operative time, and the length of hospitalization, which make rehabilitation easier and faster.^[11,12]^ Further, image navigation systems can facilitate insertion of the pedicle screw and minimize misplacement.^[13]^

However, critical and substantial evaluation of percutaneous pedicle screw fixation procedures in thoracolumbar fracture is scant, and few randomized studies have confirmed the benefits of using such techniques in spinal trauma cases. No study to our knowledge has analyzed the utilization of percutaneous techniques in traumatic thoracolumbar fractures compared with open techniques. Therefore, we performed this meta-analysis to obtain a more comprehensive conclusion on comparing the feasibility, safety and efficacy of percutaneous transpedicular screw system versus standard open procedures.

## 2 Materials and methods

### 2.1 Search strategy

To get thorough information about published studies, we conduct a PubMed, Medline, Embase, ScienceDirect, OVID, and the Cochrane CENTRAL database search and library search (Preferred Reporting Items for Systematic Reviews and Meta-Analyses) for relevant published studies from their inception to November 2014. Moreover, searches were conducted by searching the WHO International Clinical Trials Registry Platform, UK National Research Register Archive and Current Controlled Trials for unpublished studies and conference proceedings were also searched. The following search terms were used to maximize the search specificity and sensitivity: open pedicle screw fixation, percutaneous pedicle screw fixation, minimally invasive surgery, thoracolumbar fractures, thoracic vertebrae, lumbar vertebrae, bone screws, and spinal fractures.

We made restrictions on the publication language and all included studies were published in English. And the reference list of all the studies were examined to make sure there were no initially omitted studies.

### 2.2 Inclusion criteria

Studies were considered as eligible for inclusion when they met the following criteria:

Study design: Interventional studies as randomized controlled trials (RCTs) or controlled clinical trials and observational studies as cohort or case–control studies.
Population: Patients in all included studies were need posterior screw fixation in thoracolumbar junction for treatment of fractures.
Intervention: Percutaneous posterior screws fixation.
Comparator: Open posterior screws fixation.
Outcomes: The outcomes including at least one of reported operative time, subjective pain perception, blood loss, quality of life, restoration of the spinal column, and adverse events

### 2.3 Eligibility criteria

We excluded patients from this meta-analysis with following eligibility criteria: If they were not performed screws fixation for fractures of vertebra, but for some other diseases (i.e., degenerative spine diseases or neoplastic etiology). If they had a history of spinal surgery before and required direct spinal canal decompression due to neurologic deficits, the Traditional Chinese Medicine Hospital of Xinjiang Medical University has approved the study.

### 2.4 Study selection

**Figure 1:**
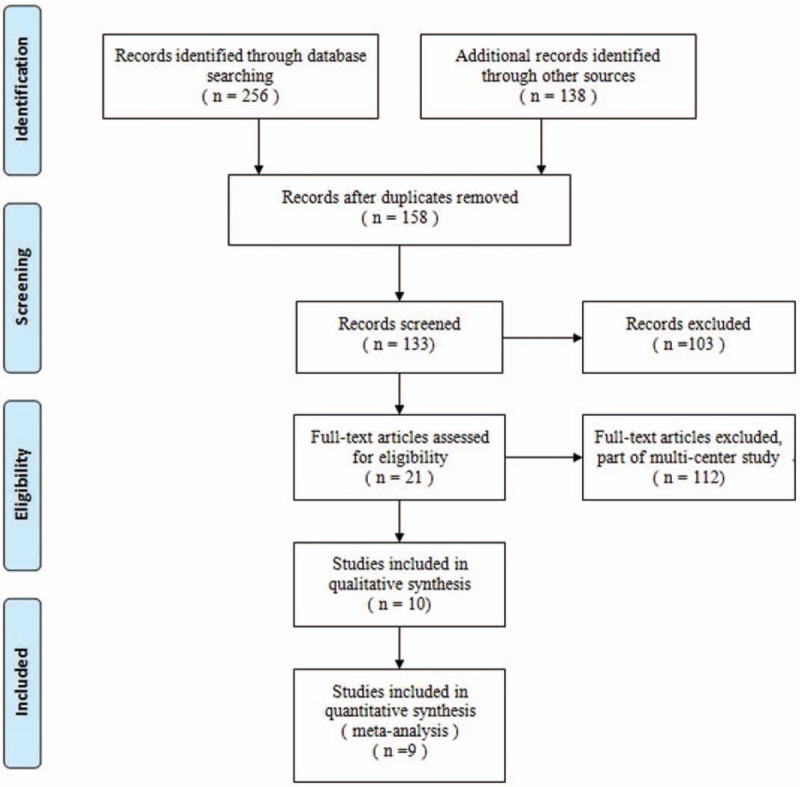
The study selection and inclusion process.

Two reviews independently screened the titles and abstracts of articles based on the eligibility criteria. When the studies met the inclusion criteria, the full text would be intensively read. If the citation could not be excluded immediately, disagreements were resolved by consensus with the senior investigator (Ma. XL.) (Fig. 1).

### 2.5 Data extraction

Two reviews (Han.Z. and Wang.X.) independently extracted the following data from each study using a standard data extraction form including the title, authors, study design, sample size, age, gender, techniques, duration of follow-up, and outcome parameters. Authors of the studies would be contacted for missing data or further information when its necessary. The extracted data were rechecked for accuracy or against the inclusion criteria by Ma.XL. (Table 1).

**Table 1:**
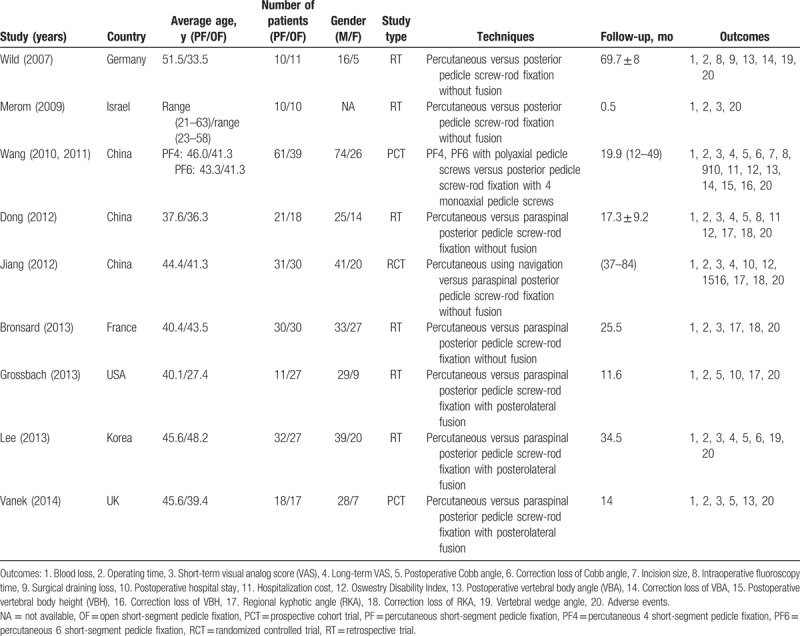
Characteristics of included studies.

### 2.6 Outcomes

The primary outcomes included visual analog scale (VAS), intraoperative blood loss, operative time, postoperative Cobb angle, postoperative vertebral body angle (VBA), and adverse events (i.e., peri-implants or superficial soft tissue infection, persistent back pain, and screw failure). The following items were included as secondary outcomes: intraoperative fluoroscopy time, surgical draining loss, postoperative hospital stay, hospitalization cost, Oswestry Disability Index (ODI), correction loss of Cobb angle, correction loss of VBA, postoperative vertebral body height (VBH), correction loss of VBH, regional kyphotic angle (RKA), correction loss of RKA, and vertebral wedge angle (VWA). “Short-term” was defined as occurring within 3 months and “long-term” as occurring after 1 year or more. If no data were reported for a specified time, we selected the closest measurements for pooling purposes.

### 2.7 Quality assessment

The methodological quality of all included studies was assessed by 2 authors (Han.Z. and Wang.X.). Any disagreements were resolved by discussion and a third author (Ma. XL.) was the adjudicator when no consensus could be achieved. For RCTs, the Cochrane Handbook for Systematic Reviews of Interventions 5.0 were used which included 7 aspects: details of randomization method; allocation concealment; blinding of participants and personnel; blinding of outcome assessment; incomplete outcome data; selective outcome reporting; and other sources of bias, to provide a qualification of risk of bias.^[14]^ For non-RCTs, the Methodological Index for Non-Randomized Studies (MINORS) scale was used for assessing methodological quality.^[15]^

### 2.8 Data analysis

Extracted data were pooled for this meta-analysis using Review Manager software (RevMan Version 5.2; The Nordic Cochrane Center, The Cochrane Collaboration, Copenhagen, Denmark). For continuous outcomes, such as VAS and intraoperative blood loss, the means and standard deviations were pooled to a weighted mean difference (WMD) and 95% confidence interval (CI). Risk ratios (RRs) and 95% CI were used to evaluate the dichotomous outcomes, such as the incidence of adverse events. Because the pooled WMD was calculated reality on the rule of intention to treat so that the dropout rate was not considered. The inverse variance and Mantel–Haenszel techniques were used to combine separate statistics and *P* < .05 was considered to be statistically significant.

Statistical heterogeneity was assessed using Q statistics. An I^2^ statistic value of 50% was considered suggestive substantial heterogeneity, referred to use a random-effect model. Otherwise, a fixed-effect model was used for the analysis. In the presence of heterogeneity, we performed sensitivity analyses to explore possible explanations for heterogeneity and to examine the influence of various exclusion criteria on the overall risk estimate. Subgroup analysis was performed to assess the outcomes at different study design.

## 3 Results

### 3.1 Search results

A total of 256 studies were preliminarily reviewed, of which 9 studies^[16–25]^ fulfilled the eligibility criteria. One of the included studies had been published into 2 papers^[23,24]^ by different period follow-up. These studies included 1 RCT,^[20]^ 1 prospective cohort studies, and 5 retrospective cohort studies. In total, 433 patients were included in the 9 studies.

### 3.2 Quality assessment

**Figure 2:**
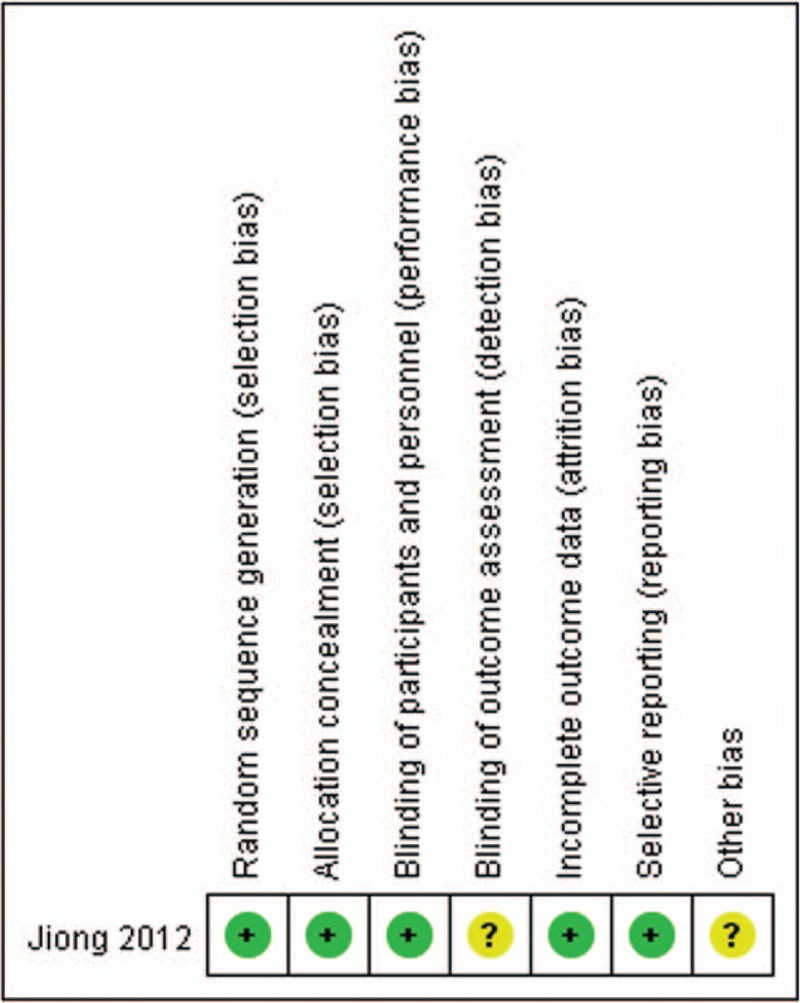
The methodological quality of the randomized controlled trials.

Among the 9 included studies, the samples were small ranging 35 to 68 participants. And only 1 RCT had a low risk of bias, and the remaining 8 non-RCT studies had^[16,22–24]^ a high risk of bias resulting from study design limitations. The methodological quality of the RCTs is shown in Fig. 2. In contrast to the RCTs, The MINORS quality scores of the non-RCTs are presented in Table 2. The mean score was 13.5 (range, 12–16), which corresponded to a 56% score. This result manifested that the evidence base of this meta-analysis has considerable variability.

**Table 2:**
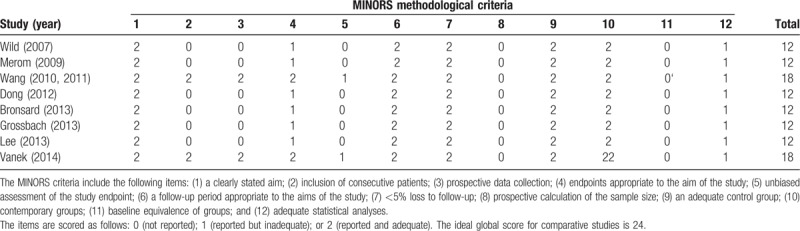
The study designs and MINORS appraisal scores for the nonrandomized controlled trials.

### 3.3 Demographic characteristics

The patients’ characteristics were comparable within each study group and 433 patients eligible for inclusion in total and the average length of follow-up ranged from 0.5 to 69.7 months. One study^[24]^ had 2 different percutaneous groups including percutaneous short-segment 4 pedicle screw fixation (PF4) group and percutaneous short-segment 6 pedicle screw fixation group (PF6) group and we divided this study into 2 comparisons as PF4 group versus open group and PF6 group versus open group.

### 3.4 Outcomes analysis

#### 3.4.1 Primary outcomes

##### 3.4.1.1 Intraoperative blood loss

**Figure 3:**
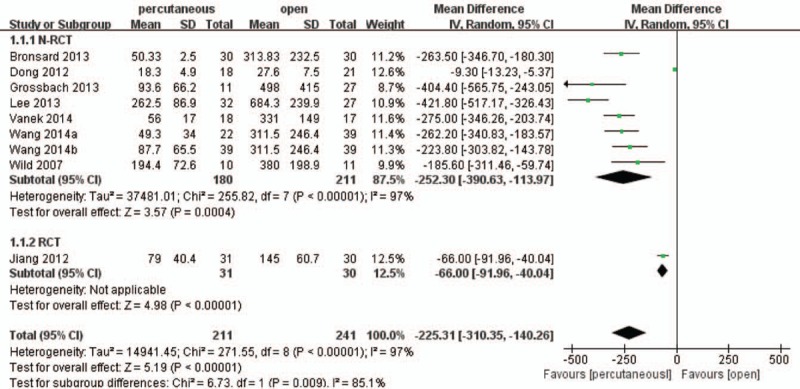
Forest plot and tabulated data illustrating the mean difference (MD) for intraoperative blood loss between percutaneous posterior pedicle screws procedures and open posterior pedicle screws procedures showing that percutaneous posterior pedicle screws procedures has a better arm blood loss and is therefore superior in this respect. CI = confidence interval, df = degrees of freedom, IV = independent variable, SD = standard deviation.

In this meta-analysis of comparative studies, 8 studies^[16,18–25]^ including 413 patients reported intraoperative blood loss and the pooled result showed percutaneous group significantly had lesser intraoperative blood loss than open group (WMD, −225.31; 95% CI, −310.35 to −140.26; *P* < .05) with significant heterogeneity (I^2^ = 97%) (Fig. 3).

##### 3.4.1.2 Operating time

**Figure 4:**
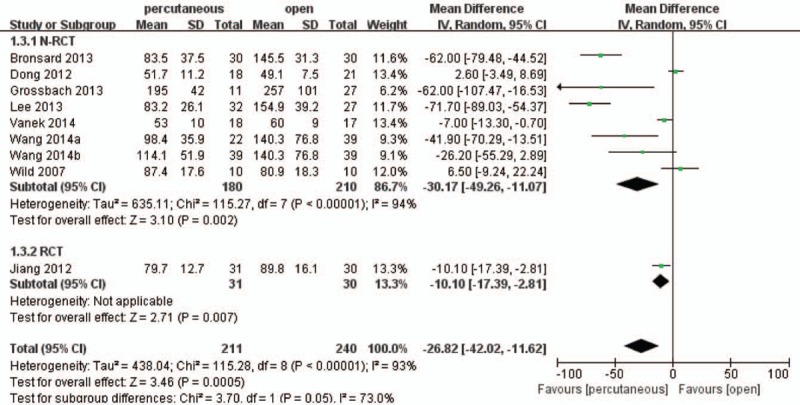
Forest plot and tabulated data illustrating the mean difference (MD) for operation time between percutaneous posterior pedicle screws procedures and open posterior pedicle screws procedures showing that percutaneous posterior pedicle screws procedures had a better arm operation time and is therefore superior in this respect. CI = confidence interval, df = degrees of freedom, IV = independent variable, SD = standard deviation.

In term of operating time, 8 studies^[16,18–25]^ including 413 patients reported intraoperative operating time and the pooled result found that percutaneous group significantly had smaller time consumption than open group (WMD, −28.62; 95% CI, −42.02 to −11.62; *P* < .05) with significant heterogeneity (I^2^ = 93%) (Fig. 4).

##### 3.4.1.3 Visual analog scale

**Figure 5:**
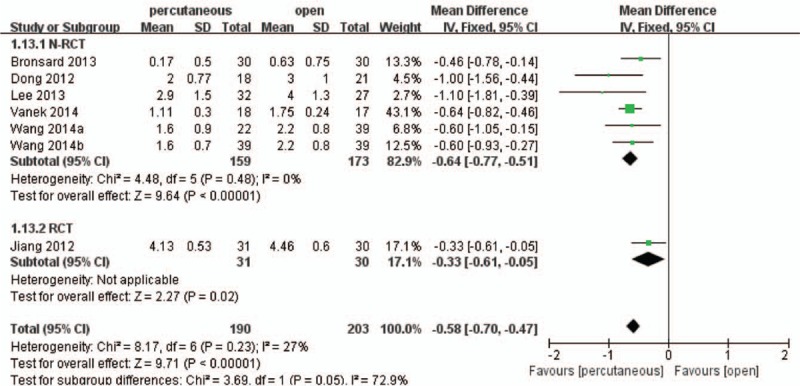
Forest plot and tabulated data illustrating the mean difference (MD) for short-term visual analog scale (VAS) between percutaneous posterior pedicle screws procedures and open posterior pedicle screws procedures showing that percutaneous posterior pedicle screws procedures had a better arm VAS and is therefore superior in this respect. CI = confidence interval, df = degrees of freedom, IV = independent variable, SD = standard deviation.

Pain was measured using VAS and was classified by the length of the follow-up period as short term and long term. Six studies^[18,20–25]^ including 354 patients reported postoperative short-term VAS and the pooled result showed that there was a significant difference between the 2 treatment groups (WMD, −0.58; 95% CI, −0.70 to −0.47; *P* < .05) with low heterogeneity (I^2^ = 27%) (Fig. 5).

**Figure 6:**
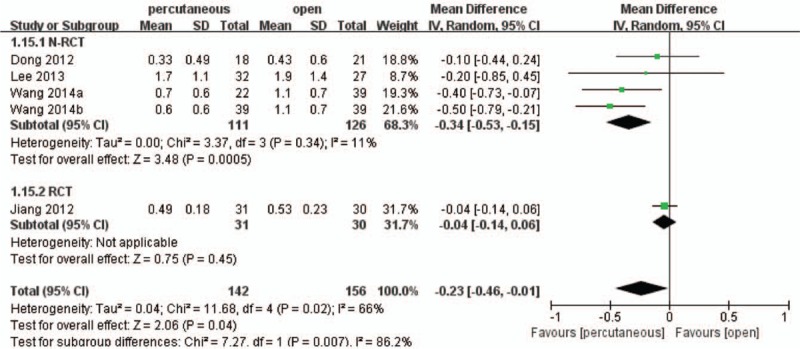
Forest plot and tabulated data illustrating the mean difference (MD) for long-term visual analog scale (VAS) between percutaneous posterior pedicle screws procedures and open posterior pedicle screws procedures showing that percutaneous posterior pedicle screws procedures had a better arm VAS and is therefore superior in this respect. CI = confidence interval, df = degrees of freedom, IV = independent variable, SD = standard deviation.

Only 4 studies^[18,20,23–25]^ including 259 patients reported intraoperative long-term VAS. The non-RCT subgroup analysis found percutaneous group was more effective than open group (WMD, −0.34; 95% CI, −0.53 to −0.15; *P* < .05) with low heterogeneity (I^2^ = 11%). However, the RCT subgroup analysis showed that there was no significant difference between the 2 treatment groups (WMD, −0.04; 95% CI, −0.14 to 0.06; *P* > .05). For total, the pooled result demonstrated that percutaneous group had a more significant effect on relieving pain compared with open group (WMD, −0.23; 95% CI, −0.46 to −0.01; *P* < .05) with significant heterogeneity (I^2^ = 66%) (Fig. 6).

##### 3.4.1.4 Postoperative Cobb angle and VBA

**Figure 7:**
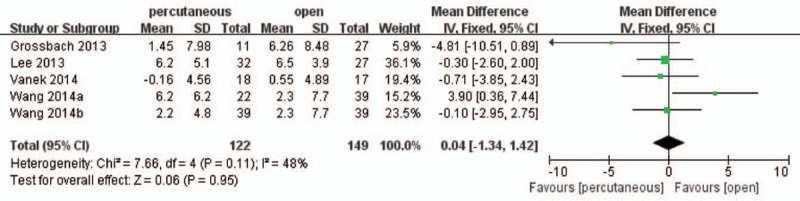
Forest plot and tabulated data illustrating the mean difference (MD) for postoperative Cobb angle between percutaneous posterior pedicle screws procedures and open posterior pedicle screws procedures showing that there is no significant difference of postoperative Cobb angle between 2 interventions. CI = confidence interval, df = degrees of freedom, IV = independent variable, SD = standard deviation.

Evidence from 4 studies^[19,21–24]^ involving 271 patients reported postoperative Cobb angle and the pooled result illustrated that there was no significant difference between the 2 treatment groups (WMD, 0.04; 95% CI, 1.34–1.42; *P* > .05) with medium heterogeneity (I^2^ = 48%) (Fig. 7).

**Figure 8:**
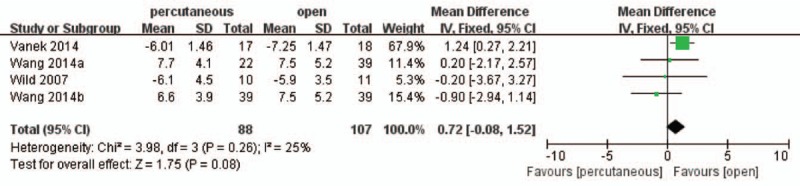
Forest plot and tabulated data illustrating the mean difference (MD) for postoperative vertebral body angle (VBA) between percutaneous posterior pedicle screws procedures and open posterior pedicle screws procedures showing that there is no significant difference of postoperative Cobb angle between 2 interventions. CI = confidence interval, df = degrees of freedom, IV = independent variable, SD = standard deviation.

Moreover, 3 studies^[16,22–24]^ involving 156 patients reported postoperative VBA and the pooled result showed that there was no significant difference between the 2 treatment groups (WMD, 0.72; 95% CI, −0.08 to 1.52; *P* > .05) with medium heterogeneity (I^2^ = 25%) (Fig. 8).

##### 3.4.1.5 Adverse events

**Figure 9:**
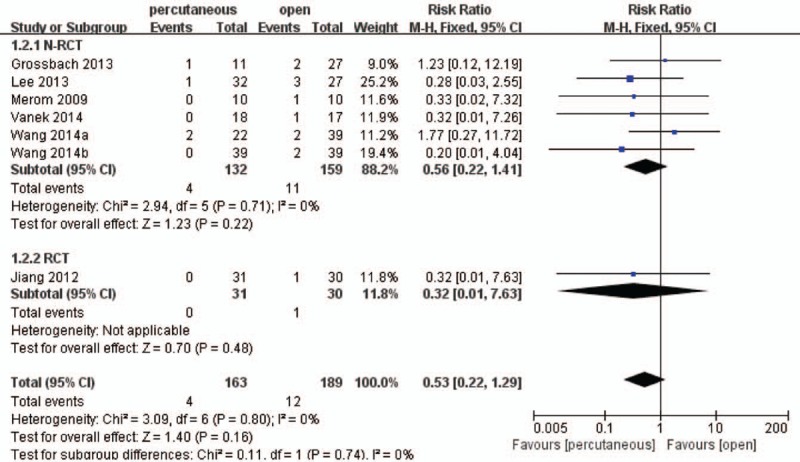
Forest plot and tabulated data illustrating the risk ratio (RR) for adverse events between percutaneous posterior pedicle screws procedures and open posterior pedicle screws procedures showing that there is no significant difference of adverse events between 2 interventions. CI = confidence interval, df = degrees of freedom, M–H = Mantel–Haenszel statistical method.

Six studies^[17,19–24]^ including 313 patients described the adverse events such as superficial infection, poor wound healing, and screw failure, which showed there were no significant differences between 2 treatment groups (RR, 0.53; 95% CI, 0.22–1.29; *P* > .05) with no heterogeneity (Fig. 9).

#### 3.4.2 Secondary outcomes

Secondary outcomes were reported as follows: for clinical outcomes, that is, incision size,^[23,24]^ intraoperative fluoroscopy time, postoperative draining loss,^[16,23,24]^ hospital stay,^[19,20,23,24]^ hospitalization cost,^[18,23,24]^ and ODI^[18,20,23,24]^; for radiological outcomes, that is, correction loss of VBA,^[16,23,24]^ correction loss of Cobb angle,^[21,23,24]^ postoperative VBH,^[16,20,23,24]^ correction loss of VBH,^[16,20,23,24]^ correction loss of RKA, postoperative RKA,^[18–20,25]^ and VWA.^[16,21]^ From our data, the pooled result illustrated that percutaneous pedicle screws techniques reduced the incision size, postoperative drainage, and postoperative hospital stay when comparing open technique. On the contrary, there were no significant in radiological outcomes, ODI, fluoroscopy time, and hospitalization cost between 2 procedures (Table 3).

**Table 3:**
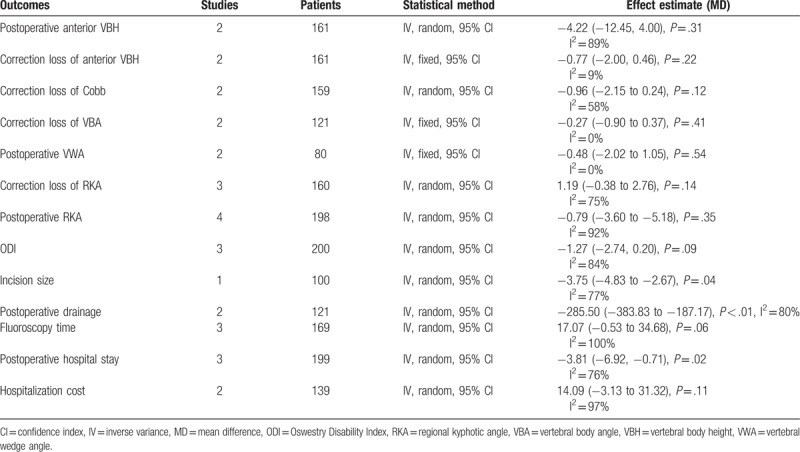
The results of secondary outcomes.

##### 3.4.2.1 Heterogeneity and sensitivity analysis

The pooled result of intraoperative blood loss, VAS, and operating time were displayed great heterogeneity in this meta-analysis and interstudy heterogeneity was not significant after dropping the most weighted study.

A sensitivity analysis was conducted after 1 RCT,^[20]^ were excluded, and no contradictory significant differences were observed in the results of the sensitivity analysis compared to the previous analysis. We therefore performed a heterogeneity interstudy, and the result indicated confidence in the conclusions of this study.

##### 3.4.2.2 Publication bias

Fewer than 10 studies were included, so we did not perform an assessment of publication bias using a funnel plot diagram.

## 4 Discussion

Management of thoracolumbar fractures remains challenging and controversial, and there is still little evidence and poor consensus focusing on the optimal technique. In the world of spine surgery, the field of minimally invasive spinal surgery has emerged within the past 2 decades as a promising novel method for thoracolumbar fracture patients. To the best of our knowledge, this is the first meta-analysis aimed to investigate the effectiveness of percutaneous posterior pedicle screws procedure for thoracolumbar fractures, make any definitive conclusions about improvement of clinical outcomes, radiologic outcomes, quality of life, and adverse events comparing conventional open procedure. Our studies suggest that percutaneous posterior pedicle screws procedure reduced the postoperative pain, intraoperative blood loss, operating time, hospitalization stay, incision size, and postoperative drainage significantly compared with conventional open procedure. However, there seems to no significant effect on rate of the method related complications, ODI, fluoroscopy time, hospitalization cost, and radiologic outcomes using percutaneous approach.

The methodological quality assessment identified some limitations to the current evidence bases. The majority of studies included in this study were small, retrospective cohort studies based on single-center experiences could introduce a high risk of bias. We identified only 1 relevant RCT met the predefined eligibility criteria. “Cochrane collaboration's tool for assessing the risk of bias” and the MINORS form were used to evaluate the RCTs and non-RCTs, respectively. Ultimately, we found that all of included non-RCTs had insufficient information on prospective calculation of the sample size and contemporary groups, which may induce low power when comparing the outcomes. Moreover, blinding of the participants and surgeons was not performed in all of the studies except one of the studies used the assessor blinding method. The lack of blinding may be associated with more exaggerated estimated intervention effects and had potential for type II statistical errors. In addition, confounding factors could disturb the intervention effect in the non-RCTs which lacking balanced by randomized methods. Therefore, most of the included studies had relatively high methodological assessment risks, which prone to have opposite forces on the accuracy and reliability of the pooled results.

From our review, heterogeneity may induce by the following factors: First, clinical heterogeneity may be caused by properties of perioperative management, different surgical technologies of percutaneous pedicle screws, and surgical complexity of the procedure. Second, the evaluation of functional recovery depends on type of fractures, rehabilitation program, follow-up time, and measure methods. Finally, characteristics of patients of individual studies, such as gender differences, pre-existing comorbidities, and economic condition, functional demands, may also be confounding factors toward marking systems. In addition, heterogeneity may have been caused by poor non-RCT study design, which induced greater bias risks than other study types. Although we performed subgroup analyses stratified by follow-up time and study design that cannot be completely resolved heterogeneity. Therefore, clinical heterogeneity should be considered when interpreting the findings, although the results of present meta-analysis were considered appropriate.

Our study suggests that the main advantage of percutaneous pedicle screws techniques was reduction of intraoperative blood loss, operating time, and postoperative pain. This finding is consistent with that reported in plenty of studies. Ni et al reported the percutaneous posterior fixation procedure leads to lower blood loss and shorter operative time and can be carried out without any special effort in thoracolumbar fractures without neurologic deficits.^[26]^ Schmidt et al also demonstrated that percutaneous pedicle screws procedure of the spine was associated with short operation time and nearly no blood loss in polytraumatized patients requiring damage control operation and geriatric patients with high perioperative risk.^[27]^ However, some studies advocated percutaneous transpedicular screw insertion was sometimes supposed to be a more technically demanding and time-consuming technique and the fact that this technique requires certain experience compared with the standard open technique.^[12,28]^ And it has been noted that a remarkable learning curve occurred despite improved median operation time and blood loss.^[29]^ The present results suggest that training with 3D navigation significantly improved the ability of orthopedic residents to properly drill simulated posterior screw.^[30]^ Another study also showed a significant improvement in amount of time taken, accuracy of fixation, and the number of exposures after the training on a navigation simulator system, which implied that such a learning curve could be overcame in a short time.^[31]^ In short, the shorter operation time and less intraoperative blood loss provided by percutaneous techniques were inspiring, because spinal trauma had frequently been related to multiple-organ injuries and unstable vital signs.

Hubbe et al revealed percutaneous pedicle screws fixation brought about the significant reduction of VAS scores regarding back pain during the first postoperative week for thoracic spine fractures.^[32]^ Moreover, Yang et al found percutaneous procedure as effective technique for treating thoracolumbar burst fractures with sustained reducing pain by a long-term follow-up.^[33]^ In addition, Kim et al found that percutaneous pedicle screw fixation caused less paraspinal muscle damage and muscle enzyme levels on the 1st and 7th day which may produce postoperative pain and negative effects on trunk muscle performance.^[34]^ The initial trauma caused by extensive dissection and retraction may have additional injury from a thermal effect and ischemia, leading eventually to sustained back pain or back muscle dysfunction.^[35]^ The decreased postoperative pain could potentially contain latent advantages, such as earlier mobilization, shorter recovery time and hospital stay, and less hospital cost, some of which had already displayed in our study. Therefore, our results suggest that early recovery of back muscle pain and function were associated with the use of percutaneous techniques, avoid extent of paraspinal muscle dissection might perfect the early clinical outcomes.

Although percutaneous pedicle screw fixation appeared to be more effective for clinical outcomes, there were no significant differences in the radiologic outcomes compared with conservative open techniques. Our outcomes revealed the rotational force required for fracture reduction and deformity correction cannot be achieved with currently available percutaneous instruments, which included polyaxial pedicle screws and nonadjustable pretending rods. Assaker first reported on the application of percutaneous transpedicular fixation in thoracolumbar trauma and there was no construct failure nor loosening with average loss of correction was 7.5.^[36]^ Palmisani et al also showed the percutaneous procedures provide comparable outcomes to those obtained with open procedures in terms of kyphosis correction and VBH recovering.^[37]^ Moreover, Yang et al suggest percutaneous short-segment pedicle procedure had a significant improvement of anterior VBH and mid-sagittal diameter, which was effective as an internal splint for burst vertebral body fractures to heal naturally. Further, Rahamimov et al advocated percutaneous augmented short-segment pedicle instrumentation of unstable thoracolumbar fractures could be done with deformity correction and the loss of correction was found to remain virtually unchanged in the following months.^[28]^

Our study paid attention to the postoperative complications of both percutaneous and open posterior pedicle screws placement. Varied complications were reported by included studies, such as infection, poor healing of wound, deep venous thrombosis, loosen screws, malposition, and breakage of screws were the most commonly seen complication. Currently, there seems to no significant difference in postoperative complications between percutaneous and open techniques for thoracolumbar fractures, though there was a trend that percutaneous groups resulted in less cases of complication and easier bone healing than open groups’ placement did. However, Wang et al maintained percutaneous techniques had a little insufficiency in resuming the anterior and posterior height of the fractured vertebral body, due to the healing of fractured vertebral body was critical for maintaining the stability and motion of the injured segment. And the percutaneous approach did not allow placement of cross-links, which would be the precondition for stabilization of longer ranging and seriously unstable segments. Therefore, excessive reposition maneuvers adopted by percutaneous approach were not feasible and sufficient reduction of the fracture should be achieved using optimized posture and manual reduction.^[27]^

Another obvious disadvantage of percutaneous techniques was lacking of bony fusion, which would add to the mechanical stability provided by open fixation, and percutaneous stabilization had been limited to relatively stable vertebral fractures which involving mainly bone component with a high power, although the necessity and reliability of fusion had long been a disputatious topics.^[31,38]^ Toyone et al had shown that short segment pedicle screw fixation without fusion could achieve satisfactory results for unstable thoracolumbar fractures.^[39]^ Ni et al revealed that satisfactory results could be obtained with only percutaneous fixation without either posterior/posterolateral or anterior fusion, and including dominant position as more motion segments, reduction of operative time during the surgery, fusion-related diseases, which was vital in case of multi trauma or severely injured patients.^[26]^ Kim et al also supported the increasing degree of mobility and subsequent hardware related to minimally invasive technique, which could potentially reduce the incidence rate for developing fusion-related complications such as proximal junctional kyphosis.^[38]^ Further, Wang et al concluded that short-segmental fixation without fusion for surgically treated burst fractures was satisfactory for clinical and radiographic parameters.^[23,24]^ Therefore, percutaneous procedures as a treatment for spinal injuries definitively should be considered as an opinion.

Our study had evaluated the 2 kinds of posterior pedicle screws placement with much more outcomes to achieve a comprehensive enough consensus on which 1 was the more appropriated for the treatment of thoracolumbar fractures. In our meta-analysis, we could draw a prudent conclusion from results of those outcomes that the percutaneous techniques had similar or even better therapeutic effect on thoracolumbar fracture to open techniques with significant smaller incision size, less postoperative drainage, shorter hospitalization stay, and a trend toward longer fluoroscopy time. The possible interpretation as follows: it is obvious that percutaneous approach make smaller incision size, and it might partly explain why percutaneous group had less postoperative drainage; second, postoperative muscle strength and truncal muscle performance was more integrated preserved in the percutaneous group, which lead to postoperative recovery and shorter hospital stay.^[40]^ Nonetheless, our result discovered the innate drawback of a distinctly longer fluoroscopy time in percutaneous techniques, which given to preoperative control of screw positioning and enable rapid correction. Yang et al^[33]^ recently showed that percutaneous pedicle screwing of the thoracolumbar spine involves the greatest radiation exposure of any percutaneous procedure in traumatology. That was quite a thorny problem for the surgeon, who would be exposed to excessive radiation over the span of his career and required steps to be taken to reduce this exposure. Mroz et al warned that surgeon would exceed occupational exposure limit for the eyes and extremities by placing 4854 and 6396 screws percutaneously, respectively.^[41]^ However, some other studies reported the different experience or methods to avoid radiation exposure.^[42]^ Smith et al^[43]^ claimed that the surgeon was regularly exposed, with varyingly negligible consequences depending on the organ concerned. And some studies reported a safer insertion technique of percutaneous pedicle screw using computer-assisted fluoroscopic navigation, 3-dimensional fluoroscopy, or a Bone Mounted Miniature Robotic System-based technique, aimed to reduce exposure to X-rays while also improving screw placement. Foley et al achieved 94.7% good screw positioning with no radiation to the surgeon's hands associated with computer-assisted fluoroscopic navigation in a cadaver study.^[44]^ Smith et al also recommended that computer-assisted image guidance reduced the surgeon's exposure and had a satisfactory result in terms of patient safety. These results were confirmed that use of navigation systems not only improved the quality of screw placement but also reduced exposure relative to 2D fluoroscopy.^[43]^ Therefore, percutaneous pedicle screw would undoubted be an optimized procedure for thoracolumbar fracture with the development of technology.

The limitations of this meta-analysis include the following: the statistical efficacy could be improved by including more studies, some of which failed to provide sufficient data though we attempted to contact the authors; all included studies were mostly designed non-RCTs and small samples, which were more likely to suffer from various types of bias; we made an effort to collect all relevant published reports and additional unpublished data, but it was inevitable to miss some information. Non-English publications were not included in this review may have important studies lost and publication bias from significant outcomes were more easily appeared; fracture type surgical techniques and postoperative care of patients which were important to the prognosis were different in the included trials which might obviously confound our pooled results; different duration of follow-up period among the included studies might affected the outcomes; and important outcomes such as depression (beck depression inventory) scores were not reported and may influence the low back disorders.^[45]^

## 5 Conclusion

This meta-analysis comparing percutaneous posterior pedicle screws procedures and open posterior pedicle screws procedures for treating thoracolumbar fractures. Our pooled results demonstrated that percutaneous procedures were superior in terms of the postoperative pain, blood loss, operating time, hospitalization stay, and incision size, but there seems to no significant effect on radiologic outcomes and rate of the method-related complications using percutaneous procedures compared open procedures. Based on the current results, we suggest the minimally invasive percutaneous procedures in cases with achieve satisfactory results could be replaced in many cases extensive open surgery and not increased related complications. However, further high-quality RCTs are needed to assess the long-term outcome of patients treated with percutaneous pedicle screw fixation compared with open instrumented techniques.

## Author contributions

**Data curation:** Lai-Yong Tu.

**Funding acquisition:** Lai-Yong Tu, Wen-Fei Gu.

**Investigation:** Ge Chu.

**Methodology:** En-Feng Zhang.

**Resources:** Feng Tian.

**Supervision:** Feng Tian, Zhen-Bin Wang.

**Validation:** Haer Ka.

**Visualization:** Haer Ka.

**Writing – original draft:** Lai-Yong Tu, Jiang Zhao.

**Writing – review & editing:** Jiang Zhao.
